# CAREGIVERS OF OLDER VIETNAMESE REFUGEES: FINDINGS FROM THE VIETNAMESE AGING AND CARE SURVEY (VACS)

**DOI:** 10.1093/geroni/igz038.669

**Published:** 2019-11-08

**Authors:** Christina E Miyawaki, Nai-Wei Chen, Oanh L Meyer, Mindy T Tran, Kyriakos S Markides

**Affiliations:** 1 Graduate College of Social Work, University of Houston, Houston, Texas, United States; 2 Beaumont Research Institute, Beaumont Health, Royal Oak, Michigan, United States; 3 University of California, Davis School of Medicine, Sacrameto, California, United States; 4 University of Houston, College of Natural Sciences and Mathematics, Houston, Texas, United States; 5 University of Texas Medical Branch, Galveston, Texas, United States

## Abstract

Since the fall of Saigon, over 1.3 million Vietnamese immigrated to the U.S. making Vietnamese the 4th largest Asian ethnic but most vulnerable group to disparities. There is a paucity of knowledge on the health of elders and their caregivers. The Vietnamese Aging and Care Survey (VACS) was developed, and health data on 67 caregivers were collected in Houston, Texas. Adult-child caregivers (n=44) were on average, 45.3 years old, married (64%), working (91%), female (61%) in good/excellent health (90%). Spousal caregivers (n=23) were 70.6 years-old, retired (57%), female (78%) in fair/good health (73%). Adult-child received more help (43%) than spousal caregivers (29%), however, felt more caregiver burden (p=0.01) and perceived stress (p=0.05). Living in a multi-generation household, sharing caregiving, and working may alleviate their financial burden and provide psychological support. Findings suggest healthcare professionals to encourage caregivers to utilize available culturally-relevant social services to further ease their caregiving experiences.

